# Conformational Ensemble of hIAPP Dimer: Insight into the Molecular Mechanism by which a Green Tea Extract inhibits hIAPP Aggregation

**DOI:** 10.1038/srep33076

**Published:** 2016-09-13

**Authors:** Yuxiang Mo, Jiangtao Lei, Yunxiang Sun, Qingwen Zhang, Guanghong Wei

**Affiliations:** 1 Key Laboratory for Computational Physical Sciences (MOE), State Key Laboratory of Surface Physics, and Department of Physics, Fudan University, Shanghai 200433, China; 2 College of Physical Science and Technology, Guangxi Normal University, 15 Yucai Road, Guilin, 541004, China; 3 College of Physical Education and Training, Shanghai University of Sport, 399 Changhai Road, Shanghai 200438, China

## Abstract

Small oligomers formed early along human islet amyloid polypeptide (hIAPP) aggregation is responsible for the cell death in Type II diabetes. The epigallocatechin gallate (EGCG), a green tea extract, was found to inhibit hIAPP fibrillation. However, the inhibition mechanism and the conformational distribution of the smallest hIAPP oligomer – dimer are mostly unknown. Herein, we performed extensive replica exchange molecular dynamic simulations on hIAPP dimer with and without EGCG molecules. Extended hIAPP dimer conformations, with a collision cross section value similar to that observed by ion mobility-mass spectrometry, were observed in our simulations. Notably, these dimers adopt a three-stranded antiparallel β-sheet and contain the previously reported β-hairpin amyloidogenic precursor. We find that EGCG binding strongly blocks both the inter-peptide hydrophobic and aromatic-stacking interactions responsible for inter-peptide β-sheet formation and intra-peptide interaction crucial for β-hairpin formation, thus abolishes the three-stranded β-sheet structures and leads to the formation of coil-rich conformations. Hydrophobic, aromatic-stacking, cation-π and hydrogen-bonding interactions jointly contribute to the EGCG-induced conformational shift. This study provides, on atomic level, the conformational ensemble of hIAPP dimer and the molecular mechanism by which EGCG inhibits hIAPP aggregation.

Amyloid fibrillar deposits formed by the aggregation of human islet amyloid polypeptide (hIAPP, also known as amylin) are pathological hallmark of type II diabetes^1^,^2^. hIAPP is a 37-residue peptide co-secreted with insulin by islet β-cell^3^,^4^ Increasing evidence shows that the low molecular weight soluble oligomers formed in the early stage of hIAPP aggregation are the most neurotoxic agents^5^,^6^,^7^,^8^. Understanding the aggregation mechanism and the structural nature of hIAPP aggregates is crucial for designing inhibitors that target hIAPP aggregation. Great efforts have been made during the past several years to reveal the nature of hIAPP aggregation. An earlier solid state nuclear magnetic resonance (ssNMR) study showed that the mature fibrils in the final hIAPP aggregates comprise a U-shaped strand-loop-strand structure (β-strand 1: A8-V17, loop: H18-L27 and β-strand 2: S28-Y37)^9^. Prior to fibril formation, this loop region was reported to be initially formed a parallel β-sheet structure^10^,^11^. The oligomer formation process of hIAPP was monitored by electrospray ionization-ion mobility spectrometry-mass spectrometry (ESI-IMS-MS) experiment and oligomers ranging from dimers to hexamers were detected^12^,^13^. Very recently, by exploiting bimolecular fluorescence complementation analysis method, Gazit and coworkers were able to observe the initial dimerization process of hIAPP aggregation^14^,^15^.

As the early formed oligomers are dynamic, heterogeneous and transient, it is experimentally challenging to characterize their atomic structures. Complement to experimental studies, all-atom molecular dynamics (MD) simulations can provide detailed structural information on small oligomers. All-atom explicit-solvent replica-exchange molecular dynamics (REMD) studies have been carried out to investigate the monomeric and oligomeric structures of different hIAPP fragments with amino acid (aa) sequence length ranging from 5 to 16 aa^16^,^17^,^18^,^19^. Recently, several groups have examined the conformational dynamics and the structural properties of full-length hIAPP monomer^20^,^21^,^22^,^23^,^24^,^25^,^26^ and the structural stability of preformed IAPP protofibrils^27^,^28^. Both implicit- and explicit-solvent REMD studies suggested that the monomeric hIAPP can transiently populate extended β-hairpin conformations^20^,^21^,^22^,^23^,^24^,^25^,^26^, and this β-hairpin was proposed to be the amyloidogenic precursor^21^,^22^. Consistent with this hypothesis, an all-atom implicit-solvent MD simulation study on the self-assembly of two preformed β-hairpin monomers showed that hIAPP dimers have a β-strand monomer-monomer interface^13^. Very recently, an implicit-solvent REMD simulation (using only 6 replicas) study suggested that disordered β-sheet-rich conformations were marginally populated in hIAPP(9–37) dimers and the dimer was formed through an α-helix-to-β-sheet transition mechanism^29^. So far, the equilibrated conformational ensemble of full-length hIAPP dimer on all-atom level remains to be determined.

In addition to hIAPP aggregation studies, the search for inhibitors of hIAPP aggregation is also an active research area. Particular attention has been focused on small molecules^30^,^31^,^32^. Among them, epigallocatechin-gallate (EGCG), as the most abundant biologically active compound in green tea, has been shown to produce unstructured, off-pathway oligomers and reduce the toxicity of hIAPP peptides^12^,^33^. Although several research groups have investigated the mechanism by which EGCG molecules inhibit the aggregation of Alzheimers amyloid-β (Aβ) peptide^34^,^35^ and the action modes of EGCG in remodeling the preformed hIAPP protofibrils^36^, the influence of EGCG molecules on hIAPP aggregation has not been explored at atomic-level details.

In this study, by performing 360-ns all-atom explicit-solvent REMD simulations on hIAPP dimer in the absence and presence of EGCG molecules, we have investigated the conformational ensembles of hIAPP dimer and the interaction mechanism between EGCG and hIAPP molecules. Starting from extended coil states, our simulations show that in the absence of EGCG molecules, hIAPP dimers transiently sample extended β-hairpin-containing three-stranded antiparallel cross β-sheet conformations, whereas EGCG binding abolishes this β-hairpin-containing dimers and significantly inhibits the formation of inter-peptide β-sheet. Contact probability and binding free energy calculation reveal that hydrophobic, aromatic stacking, cation-π and hydrogen-bonding interactions together play important roles on the EGCG-induced conformational shift of hIAPP dimer.

## Results

The convergence of the two REMD simulations was examined by comparing the following several parameters within two different time intervals using the 240–300 and 300–360 ns data for the hIAPP-dimer system and the 200–280 and 280–360 ns data for the hIAPP-dimer+EGCG system. Those parameters include the average probability of each dominant secondary structure, the secondary structure content of each amino acid residue, the probability density function (PDF) of Rg and H-bond number of hIAPP dimer. As discussed in Supporting Material, these four parameters within the two time intervals are quite similar for both systems (Figs S1(A–H) and S2(A–H)). We also checked the convergence of the REMD simulations by following the time evolution of temperature swapping of one representative replica in temperature space. As shown in Figs S1(I) and S2(I), the representative replica of the hIAPP-dimer/hIAPP-dimer+EGCG system visited the full temperature space several times during the 360 ns simulation, demonstrating that the replica was not trapped in one single temperature. Taken together, all these data demonstrate that the two REMD simulations are reasonably converged within 360 ns. All the simulation results presented below are based on the last 120 ns simulation data for hIAPP-dimer system and the last 160 ns simulation data for hIAPP-dimer+EGCG system at 310 K.

### Secondary structural characterization of hIAPP dimer in the absence and presence of EGCG molecules

**Figure 1:**
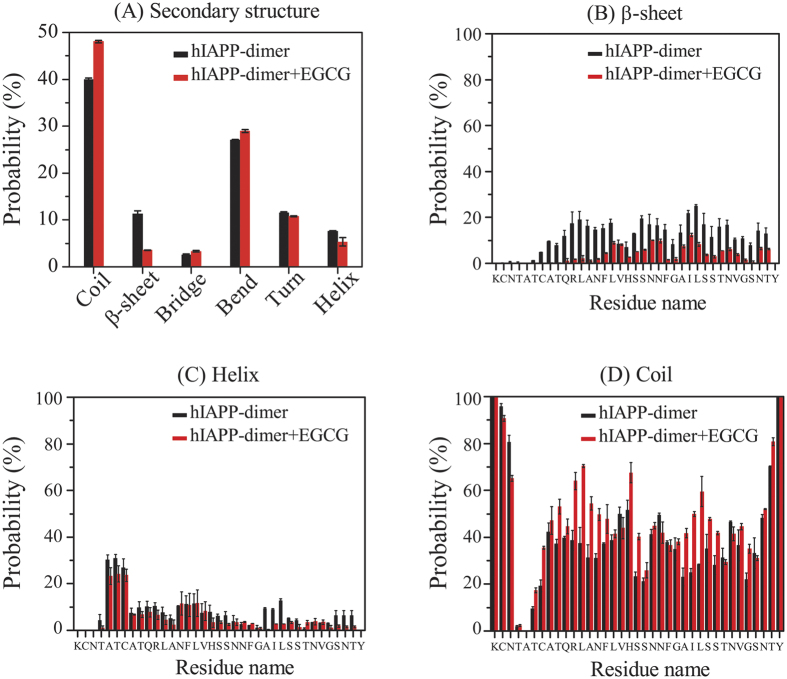
Analysis of secondary structure contents of hIAPP dimer in hIAPP-dimer and hIAPP-dimer+EGCG systems. We present the average probability of each secondary structure (**A**); the probability of β-sheet (**B**), helix (**C**) and coil (**D**) as a function of amino acid residue.

Figure 1(A) presents the secondary structure compositions of hIAPP dimer in both hIAPP-dimer and hIAPP-dimer+EGCG systems. For the hIAPP dimer without EGCG molecules, the probabilities of coil and bend structures are respectively 39.9% and 27.0%, while those of helix and β-sheet conformations are only 7.6% and 11.3%, respectively. The simulation result is consistent with the experimental observation that the majority of structural content is random coil^37^. The percentage of helix (7.6%) is also very close to the experimental value of 8% from CD spectra^37^. The β-sheet content (11.3%) is lower than that observed by experiment^37^ (21.0%), but it is higher than that (~6.5%) generated in a coarse-grained Hamiltonian-temperature REMD study for hIAPP dimer^38^. Upon addition of EGCG, the coil content is enhanced (from 39.9% without EGCG to 48.0% with EGCG), while the β-sheet content is drastically reduced (from 11.3% to 3.6%). The percentages of other secondary structures including bend, β-bridge, turn and helix do not change much. These data indicate that EGCG molecules reduce significantly the β-sheet content but enhance the random coils.

To obtain residue-based secondary structure information, we calculated the secondary structure probability as a function of amino acid residue. In the hIAPP-dimer system, residues Q10~T36 of the peptide have a relatively higher propensity to sample β-sheet conformation than other residues, in particular, residues in the FGAIL region (Fig. 1(B)) (the most amyloidogenic region of hIAPP peptide^39^). In a recent review, Strodel *et al*.^40^ discussed the effect of different force fields on the secondary structures of hIAPP dimers. It was found that the C-terminal region have a relative higher inter-peptide β-sheet propensity than the N-terminal region although the exact amino acid residues involved in the β-sheet formation differs. The N-terminal residues A5-H18 have a preference to adopt helical conformation (Fig. 1(C)). The helical region found here is consistent with the N-terminal helical region (residues A8-H18) observed in the crystal structure of hIAPP homodimer fused with a 370-residue maltose binding protein (MBP)^41^. Consistently, a recent all-atom REMD study on hIAPP(11–25) monomer identified a helical region spanning residues A13-V17^16^. In the presence of EGCG molecules, the β-sheet content in the N-terminal region involving residues A8-F15 almost disappears and that in the C-terminal region spanning residues L16-T36 is greatly reduced. The helix content keeps almost the same (Fig. 1C), while the coil conformation is enhanced for both the N-terminal residues T9-S19 and the C-terminal residues A25-G33 (Fig. 1(D)).

### An extended-β-hairpin-containing three-stranded antiparallel cross β-sheet is transiently populated in the hIAPP dimer, but it is abolished by EGCG binding

**Figure 2:**
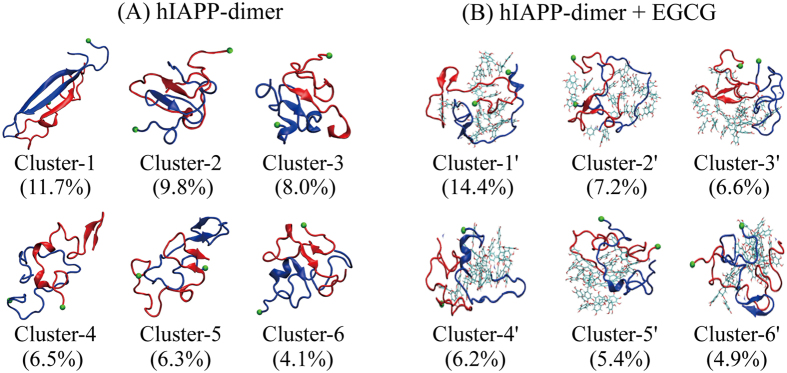
Representative conformations of the top six most-populated clusters of hIAPP dimer in hIAPP-dimer (**A**) and hIAPP-dimer+EGCG (**B**) systems. The corresponding population of each cluster is given in the parentheses. The two hIAPP chains are colored in blue and red, respectively. The EGCG molecules are presented by bond representation. The green bead represents the C_α_ atom of K1 residue of each hIAPP chain.

To investigate the three-dimensional (3D) structures of hIAPP dimer with and without EGCG molecules, we performed cluster analysis for the dimer conformations generated in both hIAPP-dimer and hIAPP-dimer+EGCG systems. With a chain-independent main-chain RMSD cutoff of 3.5 nm, the dimer conformations in the two systems are separated into 70 and 61 clusters, respectively. The centers of the top six most-populated clusters and their populations are shown in Fig. 2. These clusters represent 46.4%/44.7% for hIAPP dimer in hIAPP-dimer/hIAPP-dimer+EGCG system. In the absence EGCG molecules, the hIAPP dimer in the first cluster contains a three-stranded cross β-sheet consisting of a β-hairpin structure formed by one chain and a β-strand by the other chain. The other five clusters comprise mostly disordered coil-rich states, in which short intra-peptide and inter-peptide β-sheets are often observed (Fig. 2(A)). In the presence of EGCG molecules, the hIAPP dimer mainly adopts coil-rich conformations, in which inter-peptide β-sheets are completely lost and intra-peptide β-sheets become much shorter (Fig. 2(B)). These results demonstrate that EGCG molecules suppress completely both the β-hairpin-containing three-stranded antiparallel β-sheets and the inter-peptide β-sheets.

**Figure 3:**
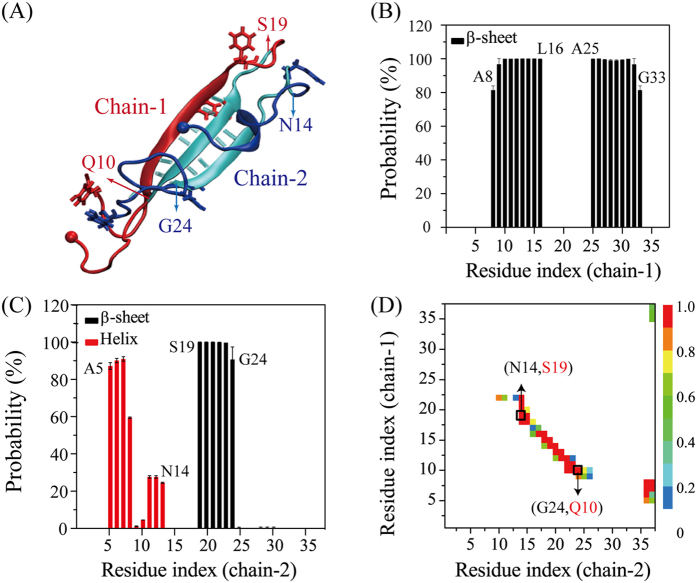
Atomic structure characterization of hIAPP dimers in cluster-1. (**A**) A representative structure of hIAPP dimer with the aromatic rings of F15, F23 and Y37 in bond representation and the hydrogen bond in cyan line. Chain-1 and chain-2 in the dimer are colored respectively in red and blue. Residues Q10-S19 in chain-1 and residues N14-G24 in chain-2 have relatively higher contact probabilities and they are colored in cyan. The C_α_ atom of K1 residue of each hIAPP chain is represented by a bead. The β-sheet and helix probabilities for each residue in chain-1 and chain-2 are given in (**B**) and (**C**), respectively. (**D**) The inter-chain MC-MC contact probability map, showing that residues Q10-S19 in chain-1 have relatively strong atomic contacts with residues N14-G24 in chain-2.

Cluster analysis is based on solely RMSD calculation, thus conformations belonging to the same cluster may not have the same secondary structure composition. To determine how many conformations in Cluster-1 that contain the aforementioned three-stranded antiparallel β-sheet structure (Fig. 3(A)), we calculated the residue-based β-sheet and helix probabilities for each peptide chain (labelled as chain-1 and chain-2) using all of the conformations in Cluster-1. Chain-1 in almost all of the dimer conformations adopts an extended β-hairpin structure (Fig. 3(B)), and chain-2 in all of the dimers displays a β-strand conformation for residues S19~G24 (Fig. 3(C)). The N-terminal residues A5-A13 (except for T9 and Q10) in 20~90% conformations forms a short helix structure. The β-hairpin consists of an N-terminal β-strand (residues A8-L16), a loop (residues V17-G24) and a C-terminal β-strand (residues A25-G33) (Fig. 3(A,B)). The positions of β-strand regions of this hairpin strongly resemble those of the ssNMR-derived hIAPP fibrils (two β-strands: A8-V17 and S28-Y37, and a loop H18-L27)^9^. The β-hairpin structure is also quite similar to the β-hairpin (two β-strands: T9-V17 and G24-G33, and a loop H18-F23) reported previously in a combined implicit-solvent REMD simulation and IMS-MS experimental study of hIAPP monomer^21^. Based on the absence of the β-hairpin conformer in both IMS-MS experiments and REMD simulations of rat IAPP under identical conditions with human IAPP and the topologic similarities of the β-hairpin to the ssNMR-derived hIAPP fibril structure by Tycko^9^, Dupuis *et al*. proposed that the β-hairpin structure was an amyloidogenic precursor of hIAPP^21^. The observation of a β-hairpin-containing three-stranded antiparallel β-sheet in our REMD simulation of hIAPP dimer further supports this hypothesis.

To identify the dominant interaction interface between the two chains in the three-stranded β-sheet, we plotted in Fig. 3(D) the main-chain−main-chain (MC-MC) contact probability map using all conformations in Cluster-1. It is noted the probability of Cluster-1 within the two time windows (240–300 ns and 300–360 ns) is 11.9% vs 11.5%, indicating that the information extracted using clustering is converged. Figure 3(D) shows that residues Q10-S19 in chain-1 and residues N14-G24 in chain-2 have relatively higher contact probabilities, indicating strong inter-peptide MC-MC interactions. This binding interface is similar to the peptide-peptide interaction interface (residues A8-H18 and N22-S28) determined by fluorescence titration binding assays^21^. The residue-residue interaction pattern along the left diagonal of the MC-MC contact map in Fig. 3(D) suggests that the two chains have a relatively high propensity to form antiparallel β-sheets. The interaction interface (residues A8-S19 in chain-1 and N14-G24 in chain-2) (see Fig. 3(A)) between the two chains in the three-stranded β-sheet dimer is mostly located between β-strand segments (residues A8-L16 in chain-1 and S20-G24 in chain-2) (see Fig. 3(B,C)). This finding is consistent with the strand-strand interface reported in a previous implicit-solvent MD study on the self-assembly of two preformed β-hairpin monomers^13^, but contrasts with the helix-helix interface derived from the crystal structure of MBP-IAPP fusion^42^.

### EGCG shifts the equilibrium of hIAPP dimer from compact β-sheet-rich conformations to loosely packed disordered coil-rich conformations

**Figure 4:**
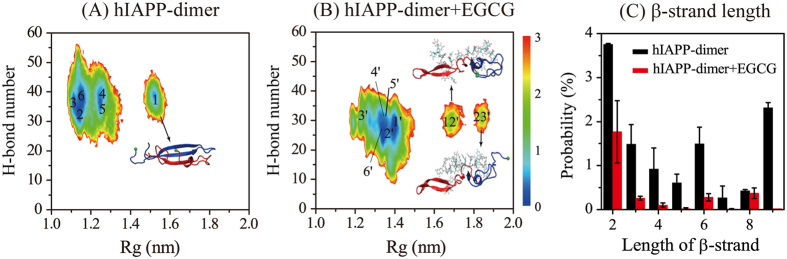
Analysis of conformational ensemble of hIAPP dimer in hIAPP-dimer and hIAPP-dimer+EGCG systems. Free energy surface (in kcal/mol) of hIAPP dimer in hIAPP-dimer (**A**) and hIAPP-dimer+EGCG (**B**) systems as a function of Rg and the total number of H-bonds (H-bond number). The locations of the top six most-populated clusters 1-6/1′-6′ are labelled in (**A**,**B**). The locations of clusters 12′ and 23′ which contains a short β-hairpin are also shown in (**B**). (**C**) Distribution of β-strand length in hIAPP dimer with and without EGCG molecules.

To probe the whole conformational space of hIAPP dimer in the absence and presence of EGCG molecules, we plotted in Fig. 4 the free energy surface of hIAPP dimer in the two different systems using –*RT* Ln *P*(H-bond number, Rg) described in Supporting Information. As shown in Fig. 4(A), there are two well-separated minimum-energy basins, centered at (Rg, H-bond number) values of (1.20 nm, 37) and (1.52 nm, 38). The deepest basin at (1.20 nm, 37) is populated by multiple conformations including compact disordered coil-rich dimers containing short β-sheet, helix and random coils. The other deepest basin at (1.52 nm, 38) corresponds to the extended three-stranded β-sheet dimer given in Fig. 3(A). Figure 4(A) shows that both compact (small Rg) and extended (large Rg) hIAPP dimers were observed in our simulations. The calculated collision cross section (CCS) of the extended dimers in Cluster-1 ranges from 1,041 to 1,401 Å^2^ (see Fig. S3). The average CCS is 1209 Å^2^, consistent with the CCS value of 1020 Å^2^ measured in recent ESI-IMS-MS experimental studies^12^,^13^. Two representative hIAPP dimers with a CCS of 1,225 Å^2^ and 1,152 Å^2^ are presented in Fig. S3(B). The free energy surface of the dimer in the presence of EGCG (Fig. 4(B)) displays a different feature from that in the absence of EGCG. There are three minimum-energy basins, centered at (Rg, H-bond number) values of (1.34 nm, 28), (1.69 nm, 30) and (1.84 nm, 32), respectively. The two basins at (Rg, H-bond number) value of (1.69 nm, 30) and (1.84 nm, 32) correspond to hIAPP dimers consisting of a β-hairpin structure (β-strand 1: residues F15-N22, β-strand 2: L27-V32) formed by one chain and a disordered coil by the other chain (see Cluster-12′ and Cluster-23′). This β-hairpin structure has different β-strand and loop regions from the one in Cluster-1 (Fig. 4(A)). Compared with the isolated hIAPP dimers, the dimers in the presence of EGCG have a decreased H-bond number and an increased Rg, implying that EGCG shifts the hIAPP dimer towards loosely packed disordered coil-rich conformations.

We also calculated the probability of β-strand length in all of the dimer conformations. Figure 4(C) shows that the probabilities of two-, three-, four-, five-, six-, and nine-residue-long β-strands in hIAPP-dimer system are 3.8%, 1.5%, 0.9%, 0.6%, 1.5%, and 2.3%, respectively, whereas they drop to 1.8%, 0.3%, 0.1%, 0.03%, 0.3% and 0.02% in hIAPP-dimet+EGCG system. The probability distribution of β-strand length, together with the free energy surface, indicate that addition of the EGCG molecules into the hIAPP dimer system leads to a reduction of the population of all lengths of β-strands and an increase of the population of disordered coil-rich dimers.

### EGCG molecules strongly block the inter-peptide hydrophobic and aromatic-stacking interactions, and alter the intra-peptide interaction patterns

**Figure 5:**
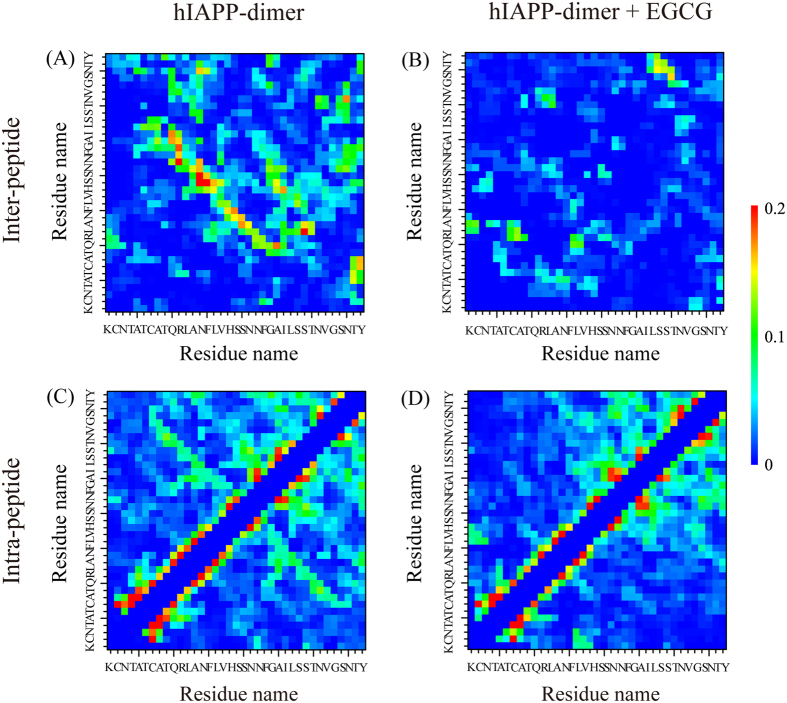
Inter-peptide (**A,B**) and intra-peptide (**C,D**) main-chain−main-chain (MC-MC) contact probability maps for hIAPP dimer with and without EGCG molecules.

To further understand the roles of EGCG on the inter-peptide and intra-peptide interactions, we calculated the MC-MC and SC-SC contact probability between all pairs of residues for the hIAPP dimer with and without EGCG. The MC-MC contact probability maps are presented in Fig. 5. The inter-peptide contact probability maps for hIAPP in the two different systems display distinct interaction patterns (Fig. 5(A,B)). In the absence of EGCG molecules, the peptide-peptide interaction interface in hIAPP dimer mainly involves residues Q10~L27 (Fig. 5(A)) that contains the FGAIL region. The relative high inter-peptide contact probabilities along the left diagonal of the contact map indicate that the two chains in hIAPP dimer are aligned predominantly in an antiparallel orientation. In the presence of EGCG molecules, the antiparallel interaction pattern disappears (Fig. 5(B)). The inter-peptide SC-SC contact probability maps for the two systems also exhibit different interaction patterns (Fig. S4(A,B)). Without EGCG, hydrophobic/aromatic residue pairs, including V17-L16 (22.9%), A13-L16 (21.7%), F23-V32 (20.1%), A13-V32 (19.0%), I26-F15 (18.9%), L16-L16 (18.4%), Y37-Y37 (16.0%), display higher contact probabilities than other residue pairs, indicating that the hydrophobic/aromatic interactions play important role in the dimerization of hIAPP. This observation is consistent with a mutagenesis study showing that three single mutations L16Q, A13E and I26D were resistant to aggregation^43^ and the triple mutant F15L/F23L/Y37L led to a decreased rate of fibrillization and a reduced toxicity of hIAPP aggregates^44^. Interestingly, we find that the residue pairs between polar and hydrophobic residues, such as S29-L12 (21.4%), Q10-I26 (19.5%), H18-A13 (19.0%), H18-L16 (18.8%) and H18-I26 (16.8%), also display higher SC-SC contact probabilities, probably due to the hydrophobic environment of these polar residues. In the presence of EGCG molecules, the SC-SC contact probabilities of all these residue pairs are greatly reduced. These data reveal that EGCG molecules significantly block inter-peptide hydrophobic and aromatic-stacking interactions.

The intra-peptide MC-MC contact probability maps are also significantly affected by the presence of EGCG (Fig. 5(C,D)). Without EGCG, a long antiparallel contact pattern exists between the N-terminal residues C7-V17 and the C-terminal residues G24-S34 (Fig. 5(C)), while it completely disappears in the presence of EGCG. Differences are also seen in the intra-peptide SC-SC contact probability maps (Fig. S4(C,D)). The contact probabilities between the N-terminal residues K1-S19 and the C-terminal residues S20-S34 were greatly reduced by the presence of EGCG, whereas those within the N-terminal region or those within the C-terminal region keep almost the same. These data demonstrate that EGCG molecules prevent primarily the long-range intra-peptide interactions between the N-terminal and C-terminal regions, while slightly affect the local interactions within each individual region. These results, together with the inhibitory role of EGCG on the inter-peptide hydrophobic/aromatic interactions (Figs 5(A,B) and S4(A,B)), explain the disappearance of three-stranded β-sheet conformations and the formation of loosely packed coil-rich conformations seen in Fig. 4. These results also provide a molecular explanation for the experimental observation that EGCG binding prevents hIAPP from assembling into higher order oligomers and fibrils^12^,^13^.

### Dominant binding sites and binding modes of EGCG molecules with hIAPP peptides

**Figure 6:**
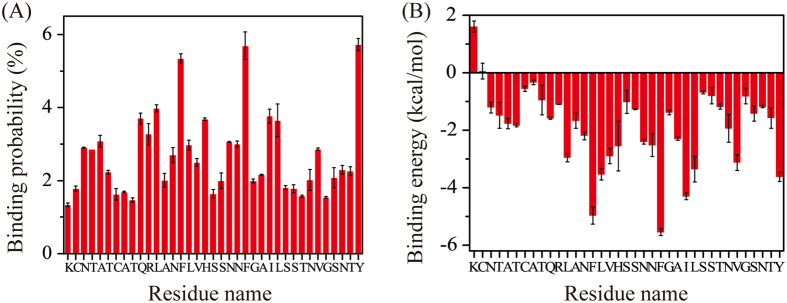
Analysis of EGCG-hIAPP binding interactions. Contact probability between heavy atoms of EGCG and each residue of hIAPP (**A**), residue-based binding free energy between EGCG and hIAPP (**B**).

We examined the dominant binding sites by calculating the contact probability and binding free energy between EGCG molecules and each amino acid residue of hIAPP. As shown in Fig. 6(A), the EGCG molecules have the highest probabilities to interact with the aromatic residues including F15 (5.3%), F23 (5.6%), and Y37 (5.7%), and have the second highest probability to bind with the hydrophobic residues including L12 (4.0%), I26 (3.8%), L27 (3.6%), and V32 (2.9%). The calculated binding free energy in Fig. 6(B) gives quantitatively the same results as Fig. 6(A). These data demonstrate that EGCG molecules preferentially interact with the aromatic and hydrophobic residues of hIAPP, revealing the crucial roles of the aromatic stacking and hydrophobic interactions. We also find that EGCG molecules display higher binding probability with polar residues Q10 (3.7%) and H18 (3.7%). H-bond calculation shows that, in more than 50% of the conformations, Q10 and H18 residues formed H-bonds with the EGCG molecules, indicating the H-bonding interaction also plays a role in EGCG-hIAPP interactions. Contact probability between the heavy atoms of EGCG and the main-chain/side-chain atoms of each residue in Fig. S5 gives quantitatively the same results. These results reveal that hydrophobic, aromatic stacking and hydrogen-bonding interactions all together play roles on the formation of loosely packed conformations of hIAPP dimer.

**Figure 7:**
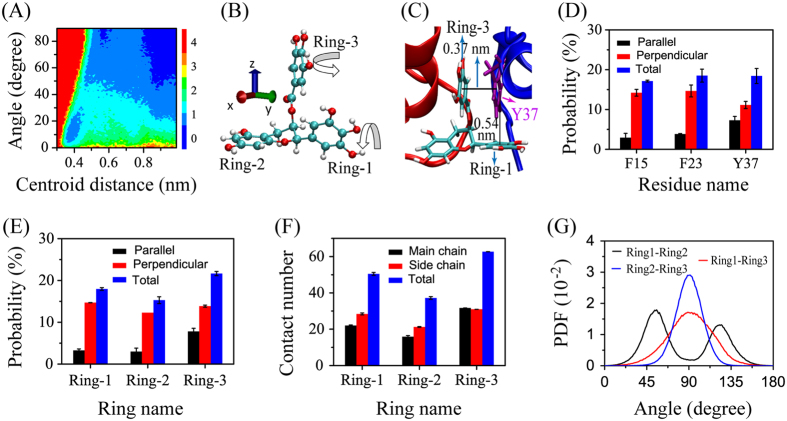
Analysis of aromatic-stacking interactions between the three aromatic residues (F15, F23 and Y37) of hIAPP and the three aromatic rings (Ring-1, Ring-2 and Ring-3) of EGCG molecules. The free energy surface as a function of the centroid distance and the angle between the side-chain rings of aromatic residues of hIAPP and the aromatic rings of EGCG molecules (**A**). A representative conformation of the EGCG molecule showing the relative position of the three phenol rings (Ring-1, Ring-2 and Rig-3). A representative snapshot showing both parallel and perpendicular stacking orientations between the aromatic ring of Y37 (purple) and the phenol ring (Ring-1 and Ring-3) of EGCG (cyan) (**C**). Formation probability for the parallel, perpendicular, and both stacking orientations between each aromatic residue of hIAPP and all of the phenol rings of EGCG molecules (**D**). Formation probability for the parallel, perpendicular, and both stacking patterns between each phenol group of EGCG molecules and all of the aromatic residues of hIAPP (**E**). Contact number between each phenol group of EGCG molecules and the main-chain, side-chain, total heavy atoms of hIAPP (**F**). Probability density function (PDF) of dihedral angles between each pair of the phenol ring (Ring1-Ring2, Ring2-Ring3, and Ring1-Ring3) of the EGCG molecules (**G**).

To better understand the aromatic interactions between hIAPP peptides and EGCG molecules, we investigated the packing orientation between the aromatic rings of residues F15, F23 and Y37 and those of EGCG. To this aim, we plotted in Fig. 7(A) the free energy surface as a function of two reaction coordinates: the centroid distance between the aromatic rings of residues F15, F23 and Y37 of hIAPP and the three phenol groups of EGCG and the dihedral angle between each pair of rings. A representative conformation of the EGCG molecule is given in Fig. 7(B), showing the relative position of the three phenol rings (Ring-1, Ring-2 and Rig-3). Three minimum-energy basins are observed in Fig. 7(A), centered respectively at (centroid distance, angle) values of (0.4 nm, 15°), (0.6 nm, 85°), and (0.9 nm, 75°). The small basin at (centroid distance, angle) values of (0.4 nm, 15°) and (0.6 nm, 85°) correspond to respectively parallel and perpendicular stacking between the aromatic rings of hIAPP residues and those of EGCG molecules. A representative snapshot is presented in Fig. 7(C), showing the parallel and perpendicular stacking between the aromatic ring of Y37 (purple) and Ring-1 and Ring-3 of an EGCG molecule (cyan). We also calculated the contact probabilities between the side-chain ring of each of the three aromatic residues and all of the aromatic rings of EGCG molecules for the parallel, perpendicular and both (parallel+perpendicular) stacking orientations (Fig. 7(D)). It can be seen that the three aromatic residues F15, F23 and Y37 make similar contributions to the perpendicular stacking interaction, while Y37 plays an important role on the parallel stacking interaction.

To identify the EGCG rings that play important roles in the aromatic stacking interaction, we calculated the formation probability of the parallel, perpendicular and both stacking orientation between each phenol group of EGCG molecules and the side-chain rings of all aromatic residues. As shown in Fig. 7(E), the gallate ester group (Ring-3) makes a greater contribution to the aromatic stacking interaction between EGCG and hIAPP than Ring-1 and Ring-2. The contact number between each phenol group of EGCG molecules and the main-chain, side-chain, total heavy atoms of hIAPP (Fig. 7(F)) shows the dominant role of the gallate ester group. This result is consistent with the experiment result that removing the gallate ester moiety leads to a less effective inhibitor for EGC^45^. It can also be seen from Fig. 7(F) that each of the three phenol rings have similar contact number with both the main chain and side chain atoms of hIAPP. To understand the dominant role of Ring-3 on EGCG-hIAPP interaction, we calculated the distribution of the dihedral angle between each pair of phenol groups (i.e. Ring1-Ring2, Ring1-Ring3, and Ring2-Ring3) of the EGCG molecule using all the conformations generated in the last 160 ns at 310 K. Figure 7(G) shows that the dihedral angle between Ring-1 and Ring-3 varies from 22° to 157°, the one between Ring-2 and Ring-3 changes from 45° to 135° (Ring-3 can freely rotate around the z-axis direction), while the one between Ring-1 and Ring-2 is restricted to 50° or 130°. These data indicate Ring-3 is the first most flexible phenol group, Ring-1 is the second most flexible phenol group, while Ring-2 is the most rigid one. This finding provides an explanation for the observation in Fig. 7(F,G) that Ring-3 has the highest contact probability/number with hIAPP peptide.

Previous studies reported that the cation-π interactions play an important role in the protein-protein and protein-ligand association^46^,^47^. We examined the cation-π interactions by analyzing the distribution of the distance between the side-chain NH_3_^+^ group of Arg and the center of each aromatic ring of EGCG. There is a sharp peak centered at 0.43 nm in the distance distribution curve (Fig. S6(A)), reflecting the existence of cation-π interactions between hIAPP and EGCG molecules. This distance is consistent with the distance of ~0.4 nm between the aromatic plane and the cation measured by experiments^48^. We give in Fig. S5(B) a representative snapshot showing the cation-π interaction observed in our simulations.

## Discussion

Previous experimental studies demonstrated that dimerization is the first step of hIAPP aggregation^15^,^41^. Thus, characterizing the structures of hIAPP dimer and the interactions with EGCG molecules is crucial for the development of drugs that target the initial dimerization step of hIAPP aggregation. In this study, by performing a 360 ns all-atom explicit REMD simulation with and without EGCG molecules, we investigated the conformational ensemble of hIAPP dimer and the key EGCG-hIAPP interactions. To the best of our knowledge, this is the first all-atom explicit-solvent REMD simulation study on the full-length hIAPP dimer and the influence of EGCG molecules. Starting from extended coil states, our simulations shows that hIAPP-dimers adopt both compact and extended conformations, and the later has a CCS similar to that measured in a recent ESI-IMS-MS experimental study^12^,^13^. The extended dimer conformation is a three-stranded antiparallel β-sheet consisting of a β-hairpin structure formed by one chain and a β-strand by the other chain. The two chains in the extended dimer have a binding interface similar to the hIAPP-hIAPP interaction interface determined by fluorescence titration binding assays^49^. This β-hairpin structure (β-strand 1: A8-L16 loop: V17-G24, and β-strand 2: A25-G33) is quite similar to the β-hairpin (β-strand 1: T9-V17, loop: H18-F23, and β-strand 2: G24-G33) reported previously in an implicit-solvent REMD simulation study of hIAPP monomer^21^. The positions of β-strand regions of this hairpin also strongly resemble those of the ssNMR-derived hIAPP fibrils (β-strand 1: A8-V17, loop: H18-L27, and β-strand 2: S28-Y37)^9^. The interaction interface between the two peptide chains in the three-stranded β-sheet dimer is almost solely between β-strand secondary structure segments (residues Q10-S19 in one chain and N14-G24 in the other chain). The observation of the β-hairpin-containing three-stranded antiparallel β-sheet in our REMD simulation supports the previous hypothesis that the β-hairpin is the amyloidogenic precursor of hIAPP^9^.

FGAIL region has been identified as the most amyloidogenic region of hIAPP peptide^39^. Our secondary structure analysis and residue-based contact probability maps show that the FGAIL region has a high tendency to form both β-sheets and inter-peptide contacts, indicating this region is of exceptional importance to the aggregation of hIAPP. Intermediates consisting of parallel β-sheets at the FGAIL region were sampled in a recent Bias-exchange metadynamics (BE-Meta) simulations^50^. However, this type of parallel β-sheets was not observed in our REMD simulations. It is noted that the BE-Meta simulations started from the U-shaped dimer conformation extracted from the ssNMR fibril structure with the FGAIL region already in parallel alignment^11^. It is interesting to know whether parallel β-sheets at the FGAIL region can be formed in larger oligomers. However, this remains to be determined.

The aromatic residues F15, F23, and F37 were shown to be very important for IAPP self-association and amyloidogenicity^51^. Interestingly, we find that the most favorable EGCG-binding residues (Q10, L12, F15, H18, F23, I26, L27, V32 and Y37) include these three residues. The binding of EGCG to these residues strongly blocks both the inter-peptide hydrophobic/aromatic interactions and the intra-peptide interactions between the N-terminal and C-terminal regions, thus abolishing the β-hairpin-containing three-stranded β-sheet conformation and shifting hIAPP dimer towards loosely packed coil-rich conformations. The hIAPP-EGCG interaction analysis demonstrates that hydrophobic, aromatic stacking, cation-π and H-bonding interactions synergistically play roles on the EGCG-induced conformational shift of hIAPP dimer. The interaction analyses between the aromatic residues (F15, F23, and Y37) (and all amino acid residues of hIAPP) and each of the phenol groups of EGCG molecules reveal that among the three phenol groups, gallate ester group of EGCG display better packing with the aromatic rings of hIAPP. This study presents an all-atom view of the conformational ensemble of hIAPP dimer and provides mechanistic insights into the inhibitory mechanism of EGCG against hIAPP aggregation.

## Materials and Methods

### hIAPP-dimer and hIAPP-dimer+EGCG systems

The sequence of hIAPP is NH_3_^+^-KCNTATCATQ^10^RLANFLVHSS^20^NNFGAILSST^30^NVGSNTY-CONH_2_, with the Cys2 and Cys7 forming a disulfide bond and the C-terminus being amidated. To mimic the experimental neutral pH condition (around pH 7.3)^52^, the N-terminus, the side chain of Lys1 and Arg11 of hIAPP are protonated (NH_3_^+^, Lys1^+^, Arg11^+^). The chemical structure of EGCG molecules (Fig. S7) taken from the ChemSpider was first optimized by Spartan’10^53^ and then energy-minimized using GAMESS software^54^. The atomic partial charges were derived using the R.E.D Ш package^55^. Other EGCG parameters were assigned based on the OPLS-AA force field^56^. The three aromatic rings in an EGCG molecule are labelled as: Ring-1, Ring-2, and Ring-3 (the gallate ester group) (Fig. S7). 12 extended states (Fig. S8) were constructed for the initial states of REMD simulations using the procedure described in Supporting Material. Each initial hIAPP dimer was placed in the center of a cubic box (6.7 × 6.7 × 6.7 nm^3^) filled with TIP4P^57^ water molecules. To approach the EGCG:hIAPP molar ratio as done experiment^12^, ten EGCG molecules were randomly placed in the simulation box (initial states was shown in Fig. S9). For simplicity, we use hIAPP-dimer and hIAPP-dimer+EGCG to label the two systems. The total numbers of atoms for the two systems are 39512 and 39122, respectively.

### REMD Simulations

REMD simulations were performed in the isothermal-isobaric (NPT) ensemble using GROMACS-4.5.3 soft package^58^, in combination with OPLS-AA force field^56^. The selection of OPLS-AA force field is based on the following reasons. First, an earlier computational study on two peptides showed that OPLS-AA generates a better balance between α-helical and β-sheet structures than AMBER, CHARMM, and GROMOS force fields^59^. Second, using the same force field, a recent study on hIAPP(11–25) peptide demonstrated that the peptide monomers transiently sampled both α-helical and β-sheet structures in solution and REMD simulations essentially reproduced the experimental H^α^ chemical shifts^16^. Finally, an earlier REMD study on hIAPP(20–29) tetramer by Mu *et al*.^19^ showed that OPLS-AA force field can generate inter-peptide β-sheets with strand orientation consistent with ssNMR data^60^. Here, REMD simulations were carried out using 48 replicas, 360 ns for each replica, at temperatures ranging from 306 to 409 K. The distribution (Table S1) of the 48 temperature was generated using an approach reported previously^61^. The LINCS^62^ and SETTLE^63^ algorithms were used to constrain the bond length of peptides and water molecules. The pressure was kept at 1 bar using the Parrinello−Rahman method^42^ with a coupling time constant of 1.0 ps. The temperature was maintained constant using a velocity rescaling coupling method^64^ with a coupling constant of 0.1 ps.

### Analysis methods

We performed the data analysis using our in-house-developed codes and the tools implemented in GROMACS-4.5.3 software package. The secondary structure of hIAPP dimer was identified using the DSSP program. The cluster analysis was performed using the Daura method with a main-chain root-mean-square deviation (RMSD) cutoff of 0.35 nm for hIAPP dimer. The chain-independent main-chain RMSD was calculated by completely neglecting the chain identifier in the coordinate file of hIAPP as the two chains are topologically identical. The two-dimensional (2D) free energy surface was constructed using –*RT* ln *P*(*x*, *y*), where *P*(*x*, *y*) is the probability of two selected reaction ordinates, *x* and *y*. The inter-peptide interactions were estimated by calculating the residue-residue contact probabilities. The binding free energies of hIAPP with EGCG were obtained by using molecular-mechanics/Poisson-Boltzmann surface area (MM/PBSA) approach (g_mmpbsa script)^65^ implemented in the GROMACS-4.6 package. The MM-PBSA approach was described in detail in a recent study^65^. The collision cross section (CCS) of the extended three-stranded β–sheet dimer structures in Cluster-1 of hIAPP-dimer was calculated using the MOBCAL software^66^,^67^ and the trajectory method^67^ which treats the target molecules as collection of atoms represented by a 12-6-4 potential.

## Additional Information

**How to cite this article**: Mo, Y. *et al*. Conformational Ensemble of hIAPP Dimer: Insight into the Molecular Mechanism by which a Green Tea Extract inhibits hIAPP Aggregation. *Sci. Rep.* **6**, 33076; doi: 10.1038/srep33076 (2016).

## Supplementary Material

Supplementary Information
