# Supplementary material for: Conformational Ensemble of hIAPP Dimer: Insight into the Molecular Mechanism by which a Green Tea Extract inhibits hIAPP Aggregation
Source: Sci Rep. 2016 Sep 13;6:33076. doi: 10.1038/srep33076 (PMC5020610; doi:10.1038/srep33076)
Supplement: Supplementary Information [file srep33076-s1.pdf]

SUPPORTING INFORMATION

Conformational Ensemble of hIAPP Dimer: Insight into the Molecular Mechanism by which a Green Tea Extract Inhibits hIAPP Aggregation

Yuxiang Mo,[1,2] Jiangtao Lei,[1] Yunxiang Sun,[1] Qingwen Zhang[3] and Guanghong Wei[1]

[1]Key Laboratory for Computational Physical Sciences (MOE), State Key Laboratory of Surface Physics, and Department of Physics, Fudan University, Shanghai 200433, China.

[2]College of Physical Science and Technology, Guangxi Normal University, 15 Yucai Road, Guilin, 541004, China.

[3]College of Physical Education and Training, Shanghai University of Sport, 399 Changhai Road, Shanghai 200438, China

This material contains details of REMD simulation and data analysis, convergence check of REMD simulations, one supplemental table and nine supplemental figures.

The coordinate files for the dimers with and without EGCG will be available free of charge upon Email to ghwei@fudan.edu.cn.

Simulation details.

To avoid any bias of initial secondary structure on the simulation results, we heated the hIAPP monomer taken from protein data bank (PDB id: 2L86) at a high temperature of 750 K to generate extended coil states of hIAPP. Then we randomly selected 24 random coil conformations from the high-temperature MD-generated hIAPP monomers. We placed each two conformations in turn in a perpendicular orientation to avoid bias in favor of parallel or antiparallel β-sheet alignments during REMD simulations. The minimum distance between the two hIAPP chains is 1.0 nm. Thus we obtained 12 initial states of hIAPP dimer for REMD simulations. The minimum distance from the hIAPP to the edge of water box is 0.8 nm. Counterions (Cl^-) were added to neutralize

the two systems. The integration time step of REMD simulation is 2 fs. Each replica was run for 360 ns, giving an accumulated simulation time of 17.28 μs for each system. The acceptance ratio for each REMD run is ~16% for both systems. Electrostatic interactions were calculated using the particle mesh Ewald method with a real space cutoff of 1.0 nm. The van der Waals interactions were calculated using a cutoff of 1.4 nm. The solute and solvent were separately coupled to external temperature and pressure baths.

Analysis details.

The two-dimensional (2D) free energy surface was constructed using $-RT \ln P(x, y)$, where $P(x, y)$ was the probability of two selected reaction ordinates, x and y. The x and y coordinates used in this study include: 1) the radius of gyration (Rg) and the number of hydrogen bonds (H-bonds) (including intra- and inter-molecular H-bonds) of hIAPP dimer; 2) the centroid distance and the orientation between the aromatic rings of F15, F23, and Y37 residues and the three rings of EGCG molecules. An H-bond was considered as formed if the N…O distance is less than 0.35 nm and the N-H…O angle is greater than 150°. A contact was defined when the aliphatic carbon atoms of two non-sequential side-chains (or main-chains) come within 0.54 nm or any other atoms of two non-sequential side-chains (or main-chains) lie within 0.46 nm. The VMD program was used for graphical structure analysis.

Convergence check of REMD simulations.

The average coil/β-sheet probabilities over all residues within the two time intervals are 39.6/12.0% versus 40.3/10.6% for the hIAPP-dimer system and 47.8/3.5% versus 48.3/3.6% for the hIAPP-dimer+EGCG system (Fig. S1 (A) and Fig. S2 (A)). The bend, turn and helix also have similar average probability within the two time intervals. The secondary structure contents for each residue within the two time intervals (Fig. S1 (B-F) for the hIAPP-dimer system and Fig. S2 (B-F) for the hIAPP-dimer+EGCG system) are also quite similar for both systems. The distributions of Rg and the total number of H-bond of the hIAPP dimer within the two time intervals also have much overlaps for both systems (Fig. S1 (H) and Fig. S2 (H)). The representative replica of the hIAPP-dimer/hIAPP-dimer+EGCG system visited the full temperature space several times during the 360 ns simulation (Fig. S1 (I) and Fig. S2 (I)), demonstrating that the replica was not trapped in one single temperature. Taken together, these data demonstrate that the two REMD simulations are reasonably converged within 360 ns.

One supplemental table

Table S1. Temperature (K) list used in the 48-replica REMD simulations of hIAPP-dimer and hIAPP-dimer+EGCG systems.

306.0	308.0	309.9	311.9	313.9	315.9	317.9	319.9
321.9	324.0	326.0	328.1	330.1	332.2	334.3	336.4
338.5	340.6	342.7	344.9	347.0	349.2	351.4	353.5
355.7	357.9	360.2	362.4	364.6	366.9	369.1	371.4
373.7	376.0	378.3	380.6	382.9	385.2	387.6	390.0
392.3	394.7	397.1	399.5	401.9	404.3	406.8	409.3

Nine supplemental figures

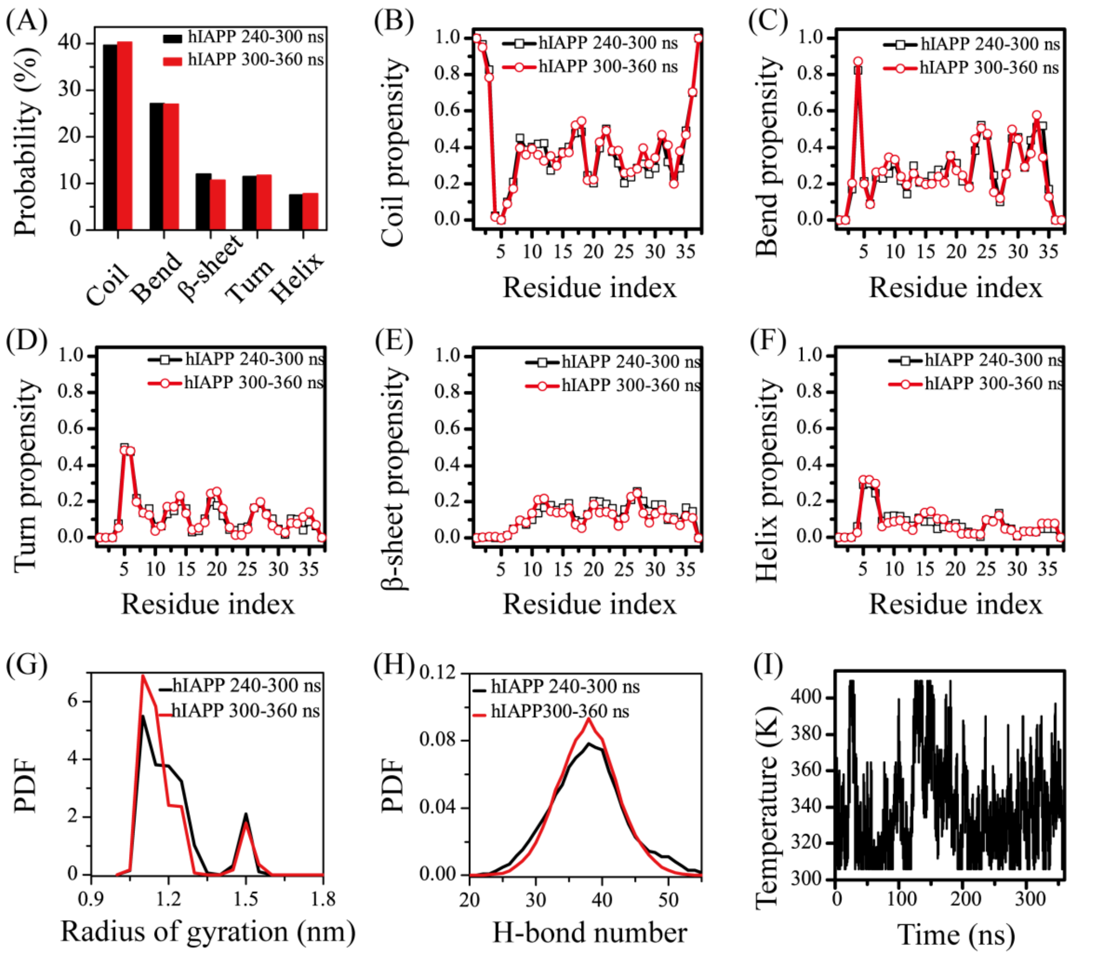

Figure S1. Simulation convergence assesments for hIAPP-dimer system using the data generated within 240-300 and 300-360 ns time intervals. We used the following several

parameters to check the convergence of the simulations: (A) the average probability of each dominant secondary structure (including coil, bend, β-sheet, turn and helix); (B)-(F) the secondary structure propensity of each amino acid residue; the probability density function (PDF) of Rg (G) and total H-bond number (H) of hIAPP dimer; (I) The time evolution of temperature swapping of one representative replica in temperature space.

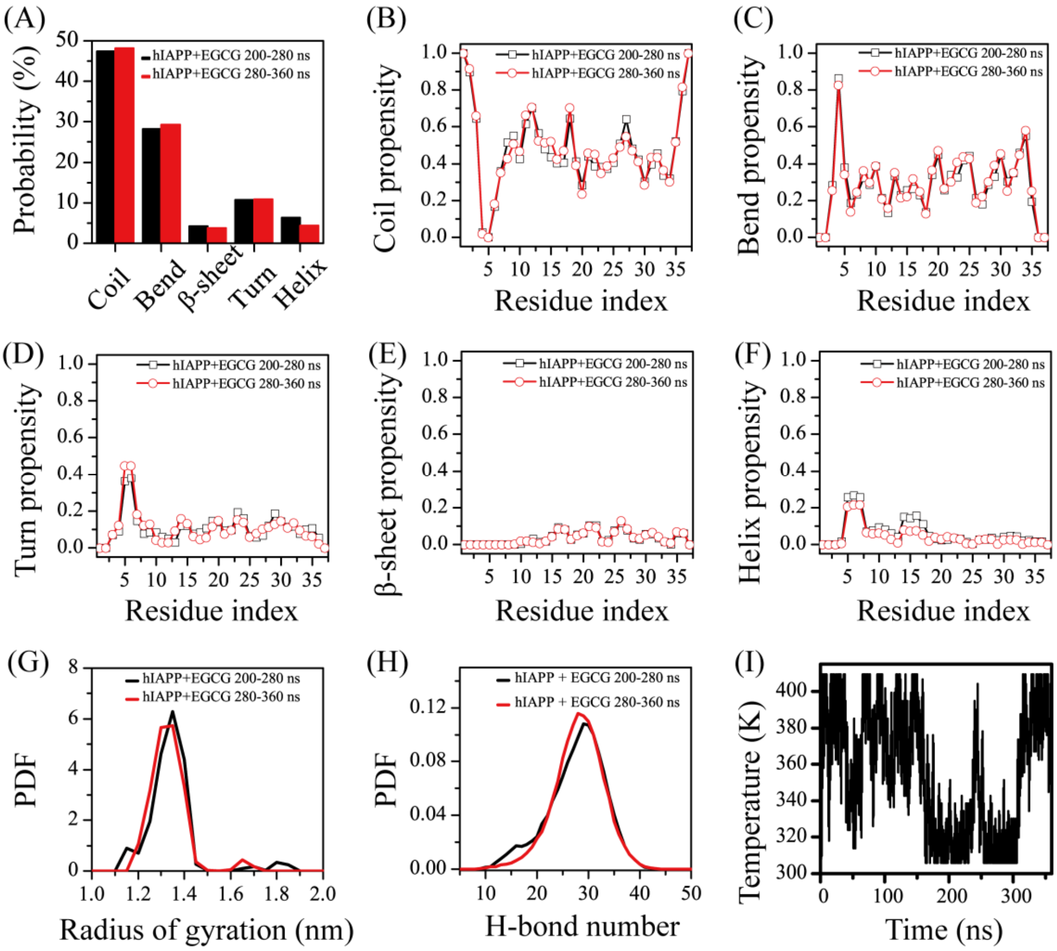

Figure S2. Simulation convergence assesments for hIAPP-dimer+EGCG system using the data generated within 240-300 and 300-360 ns time intervals. We used the following several parameters to check the convergence of the simulations: (A) the average probability of each dominant secondary structure (including coil, bend, β-sheet, turn and helix); (B)-(F) the secondary structure propensity of each amino acid residue; the probability density function (PDF) of Rg (G) and total H-bond number (H) of hIAPP dimer; (I) The time evolution of temperature swapping of one representative replica in temperature space.

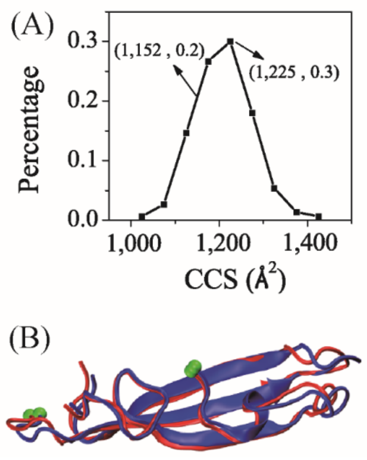

Figure S3. The percentage distribution of collision cross section (CCS) calculated using 150 frames in the first cluster of hIAPP-dimer system (A). Two representative conformations where the red one has a CCS of 1,225 Å^2 and the blue one has a CCS of 1,152 Å^2 (B).

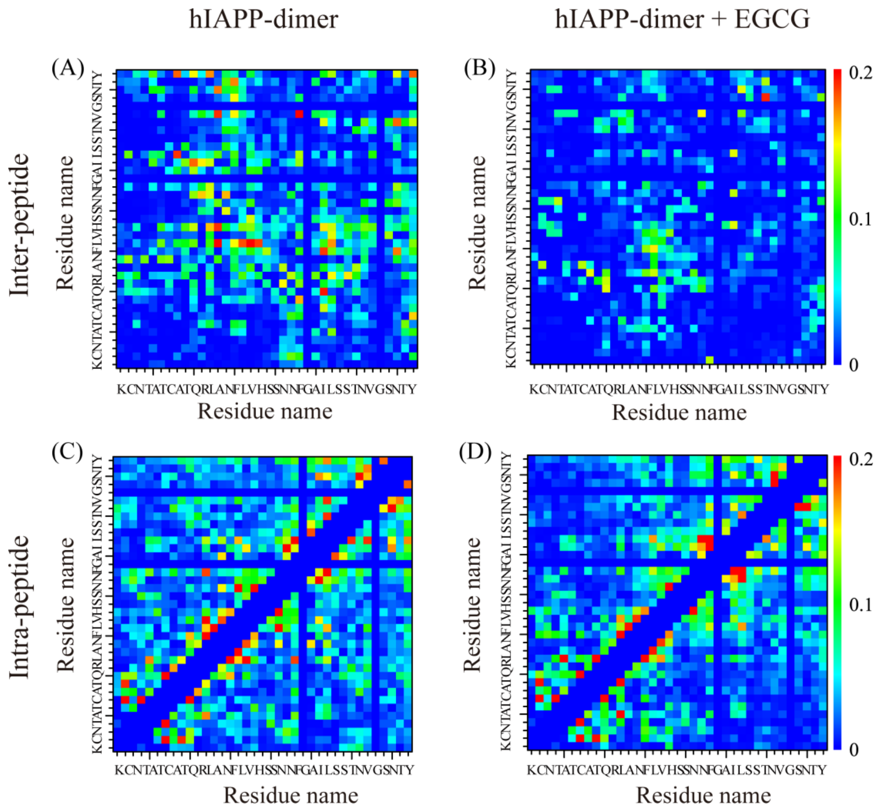

Figure S4. Inter-peptide (A,B) and intra-peptide (C, D) side-chain−side-chain (SC-SC) contact probability maps for hIAPP in hIAPP-dimer and hIAPP-dimer+EGCG systems.

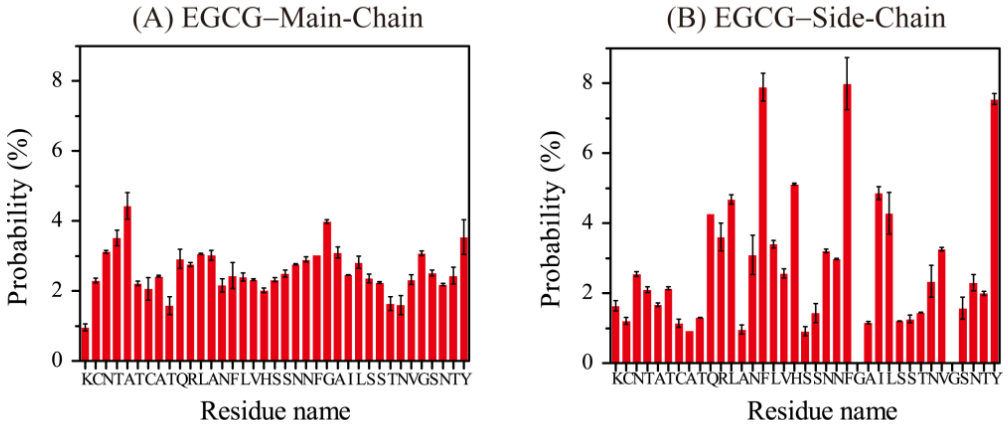

Figure S5. Analysis of contact probability between the heavy atoms of EGCG and each amino acid residue of hIAPP. Interactions of EGCG molecules with the main-chain (A) and side-chain atoms of hIAPP dimer.

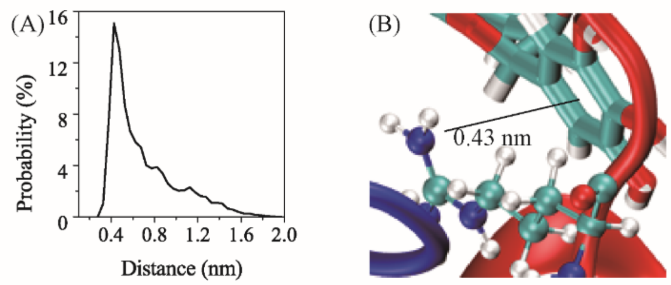

Figure S6. Analysis of the cation-π interaction. The distribution of the minimum distance between the side chain NH_3^+ group of Arg and the center of each aromatic ring (A). A representative snapshot in which the distance between the side chain NH_3^+ group of Arg11 and the ring_1 of EGCG is 0.43 nm (B). This short distance indicates the existence of cation-π interaction.

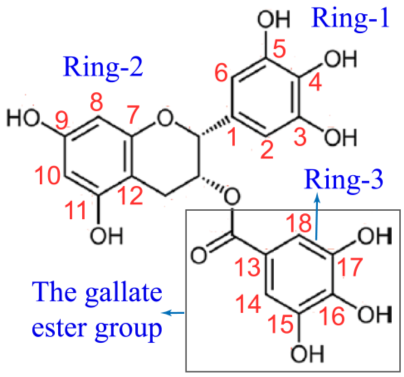

Figure S7. The chemical structure of an EGCG molecule. The red numbers label the carbon atoms in the three different aromatic rings: Ring-1, Ring-2 and Ring-3.

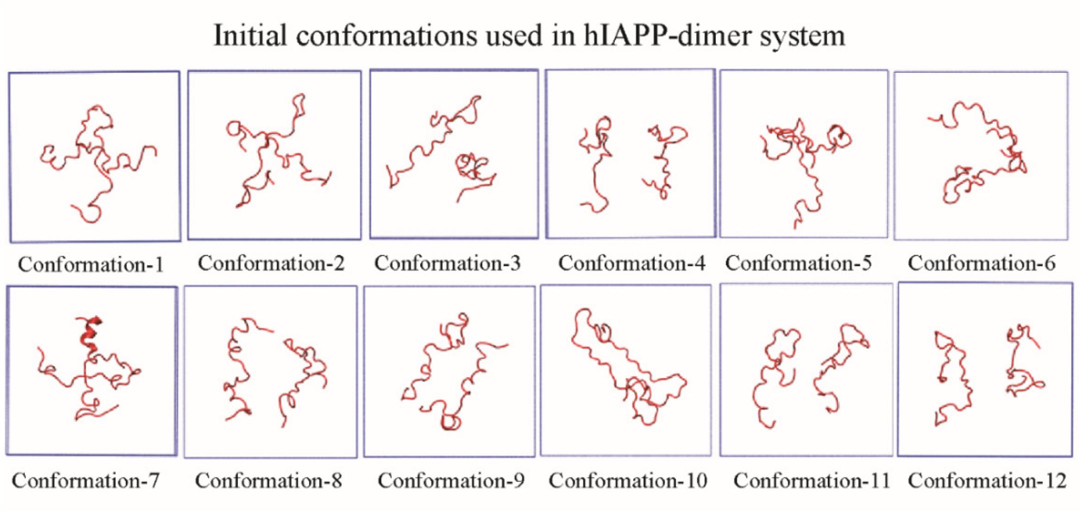

Figure S8. The 12 initial states of hIAPP-dimer system. The peptides are shown by new-cartoon representation using VMD.

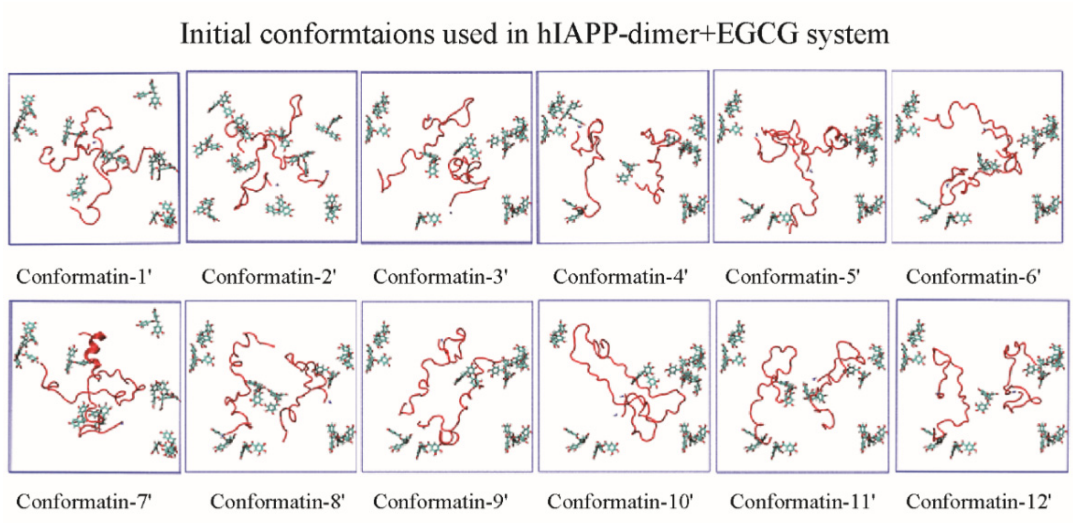

Figure S9. The 12 initial states of hIAPP-dimer+EGCG system. The peptides are shown by new-cartoon representation using VMD.
